# A Two-Moment Inequality with Applications to Rényi Entropy and Mutual Information

**DOI:** 10.3390/e22111244

**Published:** 2020-11-01

**Authors:** Galen Reeves

**Affiliations:** 1 Department of Electrical and Computer Engineering, Duke University, Durham, NC 27708, USA; galen.reeves@duke.edu; 2 Department of Statistical Science, Duke University, Durham, NC 27708, USA

**Keywords:** information inequalities, mutual information, Rényi entropy, Carlson–Levin inequality

## Abstract

This paper explores some applications of a two-moment inequality for the integral of the *r*th power of a function, where 0<r<1. The first contribution is an upper bound on the Rényi entropy of a random vector in terms of the two different moments. When one of the moments is the zeroth moment, these bounds recover previous results based on maximum entropy distributions under a single moment constraint. More generally, evaluation of the bound with two carefully chosen nonzero moments can lead to significant improvements with a modest increase in complexity. The second contribution is a method for upper bounding mutual information in terms of certain integrals with respect to the variance of the conditional density. The bounds have a number of useful properties arising from the connection with variance decompositions.

## 1. Introduction

The interplay between inequalities and information theory has a rich history, with notable examples including the relationship between the Brunn–Minkowski inequality and the entropy power inequality as well as the matrix determinant inequalities obtained from differential entropy [1]. In this paper, the focus is on a “two-moment” inequality that provides an upper bound on the integral of the *r*th power of a function. Specifically, if *f* is a nonnegative function defined on $R^{n}$ and p,q,r are real numbers satisfying 0<r<1 and p<1/r−1<q, then

(1) $$\begin{array}{c} {\left(\int f{\left(x\right)}^{r}\;dx\right)}^{\frac{1}{r}}\leq C_{n,p,q,r}\;{\left(\int {∥x∥}^{np}f\left(x\right)\;dx\right)}^{\frac{qr+r-1}{(q-p)r}}{\left(\int {∥x∥}^{nq}f\left(x\right)\;dx\right)}^{\frac{1-r-pr}{(q-p)r}}, \end{array}$$

where the best possible constant $C_{n,p,q,r}$ is given exactly; see Propositions 2 and 3 ahead. The one-dimensional version of this inequality is a special case of the classical Carlson–Levin inequality [2,3,4], and the multidimensional version is a special case of a result presented by Barza et al. [5]. The particular formulation of the inequality used in this paper was derived independently in [6], where the proof follows from a direct application of Hölder’s inequality and Jensen’s inequality.

In the context of information theory and statistics, a useful property of the two-moment inequality is that it provides a bound on a nonlinear functional, namely the *r*-quasi-norm ${∥\cdot ∥}_{r}$, in terms of integrals that are linear in *f*. Consequently, this inequality is well suited to settings where *f* is a mixture of simple functions whose moments can be evaluated. We note that this reliance on moments to bound a nonlinear functional is closely related to bounds obtained from variational characterizations such as the Donsker–Varadhan representation of Kullback divergence [7] and its generalizations to Rényi divergence [8,9].

The first application considered in this paper concerns the relationship between the entropy of a probability measure and its moments. This relationship is fundamental to the principle of maximum entropy, which originated in statistical physics and has since been applied to statistical inference problems [10]. It also plays a prominent role in information theory and estimation theory where the fact that the Gaussian distribution maximizes differential entropy under second moment constraints ([11], [Theorem 8.6.5]) plays a prominent role. Moment–entropy inequalities for Rényi entropy were studied in a series of works by Lutwak et al. [12,13,14], as well as related works by Costa et al. [15,16] and Johonson and Vignat [17], in which it is shown that, under a single moment constraint, Rényi entropy is maximized by a family of generalized Gaussian distributions. The connection between these moment–entropy inequalities and the Carlson–Levin inequality was noted recently by Nguyen [18].

In this direction, one of the contributions of this paper is a new family of moment–entropy inequalities. This family of inequalities follows from applying Inequality (1) in the setting where *f* is a probability density function, and thus there is a one-to-one correspondence between the integral of the *r*th power and the Rényi entropy of order *r*. In the special case where one of the moments is the zeroth moment, this approach recovers the moment–entropy inequalities given in previous work. More generally, the additional flexibility provided by considering two different moments can lead to stronger results. For example, in Proposition 6, it is shown that if *f* is the standard Gaussian density function defined on $R^{n}$, then the difference between the Rényi entropy and the upper bound given by the two-moment inequality (equivalently, the ratio between the left- and right-hand sides of (1)) is bounded uniformly with respect to *n* under the following specification of the moments:

(2) $$\begin{array}{cc} p_{n} & =\frac{1-r}{r}-\frac{1}{r}\sqrt{\frac{2(1-r)}{n+1}},\;q_{n}=\frac{1-r}{r}+\frac{1}{r}\sqrt{\frac{2(1-r)}{n+1}}. \end{array}$$

Conversely, if one of the moments is restricted to be equal to zero, as is the case in the usual moment–entropy inequalities, then the difference between the Rényi entropy and the upper bound diverges with *n*.

The second application considered in this paper is the problem of bounding mutual information. In conjunction with Fano’s inequality and its extensions, bounds on mutual information play a prominent role in establishing minimax rates of statistical estimation [19] as well as the information-theoretic limits of detection in high-dimensional settings [20]. In many cases, one of the technical challenges is to provide conditions under which the dependence between the observations and an underlying signal or model parameters converges to zero in the limit of high dimension.

This paper introduces a new method for bounding mutual information, which can be described as follows. Let $P_{X,Y}$ be a probability measure on X×Y such that $P_{Y|X=x}$ and $P_{Y}$ have densities f(y∣x) and f(y) with respect to the Lebesgue measure on $R^{n}$. We begin by showing that the mutual information between *X* and *Y* satisfies the upper bound

(3) $$\begin{array}{c} I\left(X;Y\right)\leq \int \sqrt{\mathrm{Var}(f(y|X))}\;dy, \end{array}$$

where $\mathrm{Var}\left(p\left(y|X\right)\right)=\int {\left(f(y|x)-f(y)\right)}^{2}\;dP_{X}\left(x\right)$ is the variance of f(y∣X); see Proposition 8 ahead. In view of (3), an application of the two-moment Inequality (1) with r=1/2 leads to an upper bound with respect to the moments of the variance of the density:

(4) $$\begin{array}{c} \int {∥y∥}^{ns}\mathrm{Var}\left(f\left(y|X\right)\right)\;dy \end{array}$$

where this expression is evaluated at s∈{p,q} with p<1<q. A useful property of this bound is that the integrated variance is quadratic in $P_{X}$, and thus Expression (4) can be evaluated by swapping the integration over *y* and with the expectation of over two independent copies of *X*. For example, when $P_{X,Y}$ is a Gaussian scale mixture, this approach provides closed-form upper bounds in terms of the moments of the Gaussian density. An early version of this technique is used to prove Gaussian approximations for random projections [21] arising in the analysis of a random linear estimation problem appearing in wireless communications and compressed sensing [22,23].

## 2. Moment Inequalities

Let $L^{p}\left(S\right)$ be the space of Lebesgue measurable functions from *S* to R whose *p*th power is absolutely integrable, and for p≠0, define

$${∥f∥}_{p}:={\left(\int_{S}{\left|f(x)\right|}^{p}\;dx\right)}^{1/p}.$$

Recall that ${∥\cdot ∥}_{p}$ is a norm for p≥1 but only a quasi-norm for 0<p<1 because it does not satisfy the triangle inequality. The *s*th moment of *f* is defined as

$$M_{s}\left(f\right):=\int_{S}{∥x∥}^{s}\;\left|f\left(x\right)\right|\;dx,$$

where ∥·∥ denotes the standard Euclidean norm on vectors.

The two-moment Inequality (1) can be derived straightforwardly using the following argument. For r∈(0,1), the mapping $f\mapsto {∥f∥}_{r}$ is concave on the subset of nonnegative functions and admits the variational representation

(5) $$\begin{array}{c} {∥f∥}_{r}=\inf\left\{\frac{{∥fg∥}_{1}}{{∥g∥}_{r^{*}}}\;:\;g\in L^{r^{*}}\right\}, \end{array}$$

where $r^{*}=r/\left(r-1\right)\in \left(-\infty ,0\right)$ is the Hölder conjugate of *r*. Consequently, each $g\in L^{r^{*}}$ leads to an upper bound on ${∥f∥}_{r}$. For example, if *f* has bounded support *S*, choosing *g* to be the indicator function of *S* leads to the basic inequality ${∥f∥}_{r}\leq {\left(\mathrm{Vol}\left(S\right)\right)}^{(1-r)/r}{∥f∥}_{1}$. The upper bound on ${∥f∥}_{r}$ given in Inequality (1) can be obtained by restricting the minimum in Expression (5) to the parametric class of functions of the form $g\left(x\right)=\nu_{1}{∥x∥}^{np}+\nu_{2}\;{∥x∥}^{nq}$ with $\nu_{1},\nu_{2}>0$ and then optimizing over the parameters $\left(\nu_{1},\nu_{2}\right)$. Here, the constraints on p,q are necessary and sufficient to ensure that $g\in L^{r^{*}}\left(R^{n}\right)$.

In the following sections, we provide a more detailed derivation, starting with the problem of maximizing ${∥f∥}_{r}$ under multiple moment constraints and then specializing to the case of two moments. For a detailed account of the history of the Carlson type inequalities as well as some further extensions, see [4].

### 2.1. Multiple Moments

Consider the following optimization problem:

$$\begin{array}{cccc} \mathrm{maximize}\; & {∥f∥}_{r}\\ \mathrm{subject}\;\mathrm{to}\; & f(x)\geq 0 & \; & \mathrm{for}\;\mathrm{all}\;x\in S\\ & M_{s_{i}}\left(f\right)\leq m_{i} & & \mathrm{for}\;1\leq i\leq k. \end{array}$$

For r∈(0,1), this is a convex optimization problem because ${∥\cdot ∥}_{r}^{r}$ is concave and the moment constraints are linear. By standard theory in convex optimization (e.g., [24]), it can be shown that if the problem is feasible and the maximum is finite, then the maximizer has the form

$$f^{*}\left(x\right)={\left(\sum_{i=1}^{k}\nu_{i}^{*}\;{∥x∥}^{s_{i}}\right)}^{\frac{1}{r-1}},\;\mathrm{for}\;\mathrm{all}\;x\in S.$$

The parameters $\nu_{1}^{*},\cdots ,\nu_{k}^{*}$ are nonnegative and the *i*th moment constraint holds with equality for all *i* such that $\nu_{i}^{*}$ is strictly positive—that is, $\nu_{i}^{*}>0\Rightarrow \mu_{s_{i}}\left(f^{*}\right)=m_{i}$. Consequently, the maximum can be expressed in terms of a linear combination of the moments:

$$∥f^{*}{∥}_{r}^{r}=∥{\left(f^{*}\right)}^{r}{∥}_{1}={∥f^{*}{\left(f^{*}\right)}^{r-1}∥}_{1}=\sum_{i=1}^{k}\nu_{i}^{*}m_{i}.$$

For the purposes of this paper, it is useful to consider a relative inequality in terms of the moments of the function itself. Given a number 0<r<1 and vectors $s\in R^{k}$ and $\nu \in R_{+}^{k}$, the function $c_{r}\left(\nu ,s\right)$ is defined according to

$$c_{r}\left(\nu ,s\right)={\left(\int_{0}^{\infty }{\left(\sum_{i=1}^{k}\nu_{i}\;x^{s_{i}}\right)}^{-\frac{r}{1-r}}dx\right)}^{\frac{1-r}{r}},$$

if the integral exists. Otherwise, $c_{r}\left(\nu ,s\right)$ is defined to be positive infinity. It can be verified that $c_{r}\left(\nu ,s\right)$ is finite provided that there exists i,j such that $\nu_{i}$ and $\nu_{j}$ are strictly positive and $s_{i}<\left(1-r\right)/r<s_{j}$.

The following result can be viewed as a consequence of the constrained optimization problem described above. We provide a different and very simple proof that depends only on Hölder’s inequality.

**Proposition** **1.** *Let f be a nonnegative Lebesgue measurable function defined on the positive reals $R_{+}$. For any number 0<r<1 and vectors $s\in R^{k}$ and $\nu \in R_{+}^{k}$, we have*

$${∥f∥}_{r}\leq c_{r}\left(\nu ,s\right)\sum_{i=1}^{k}\nu_{i}\;M_{s_{i}}\left(f\right).$$

**Proof.**  Let $g\left(x\right)=\sum_{i=1}^{k}\nu_{i}\;x^{s_{i}}$. Then, we have

$$\begin{array}{cc} {∥f∥}_{r}^{r} & =∥g^{-r}{\left(fg\right)}^{r}{∥}_{1}\leq ∥g^{-r}{∥}_{\frac{1}{1-r}}{∥\left(gf\right)}^{r}{∥}_{\frac{1}{r}}=∥g^{\frac{-r}{1-r}}{∥}_{1}^{1-r}{∥gf∥}_{1}^{r}={\left(c_{r}\left(\nu ,s\right)\;\sum_{i=1}^{k}\nu_{i}\;M_{s_{i}}\left(f\right)\right)}^{r}, \end{array}$$

where the second step is Hölder’s inequality with conjugate exponents 1/(1−r) and 1/r. □

### 2.2. Two Moments

For a,b>0, the beta function B(a,b) and gamma function Γ(a) are given by

$$\begin{array}{cc} B(a,b) & =\int_{0}^{1}t^{a-1}{\left(1-t\right)}^{b-1}\;dt\\ \Gamma (a) & =\int_{0}^{\infty }t^{a-1}e^{-t}\;dt, \end{array}$$

and satisfy the relation B(a,b)=Γ(a)Γ(b)/Γ(a+b), a,b>0. To lighten the notation, we define the normalized beta function

(6) $$\begin{array}{c} \tilde{B}\left(a,b\right)=B\left(a,b\right){\left(a+b\right)}^{a+b}a^{-a}b^{-b}. \end{array}$$

Properties of these functions are provided in Appendix A.

The next result follows from Proposition 1 for the case of two moments.

**Proposition** **2.** *Let f be a nonnegative Lebesgue measurable function defined on [0,∞). For any numbers p,q,r with 0<r<1 and p<1/r−1<q,*

$${∥f∥}_{r}\leq {\left[\psi_{r}\left(p,q\right)\right]}^{\frac{1-r}{r}}\;{\left[M_{p}\left(f\right)\right]}^{\lambda }{\left[M_{q}\left(f\right)\right]}^{1-\lambda },$$

*where λ=(q+1−1/r)/(q−p) and*

(7) $$\begin{array}{c} \psi_{r}\left(p,q\right)=\frac{1}{\left(q-p\right)}\tilde{B}\left(\frac{r\lambda }{1-r},\frac{r(1-\lambda )}{1-r}\right), \end{array}$$

*where $\tilde{B}\left(\cdot ,\cdot \right)$ is defined in Equation (6).*

**Proof.**  Letting s=(p,q) and $\nu =(\gamma^{1-\lambda },\gamma^{-\lambda })$ with λ>0, we have

$${\left[c_{r}\left(\nu ,s\right)\right]}^{\frac{r}{1-r}}=\int_{0}^{\infty }{\left(\gamma^{1-\lambda }\;x^{p}+\gamma^{-\lambda }\;x^{q}\right)}^{-\frac{r}{1-r}}\;dx.$$

Making the change of variable $x\mapsto {\left(\gamma u\right)}^{\frac{1}{q-p}}$ leads to

$${\left[c_{r}\left(\nu ,s\right)\right]}^{\frac{r}{1-r}}=\frac{1}{q-p}\int_{0}^{\infty }\frac{u^{b-1}}{{\left(1+u\right)}^{a+b}}\;du=\frac{B\left(a,b\right)}{q-p},$$

where $a=\frac{r}{1-r}\lambda$ and $b=\frac{r}{1-r}\left(1-\lambda \right)$ and the second step follows from recognizing the integral representation of the beta function given in Equation (A3). Therefore, by Proposition 1, the inequality

$${∥f∥}_{r}\leq {\left(\frac{B\left(a,b\right)}{q-p}\right)}^{\frac{1-r}{r}}\left(\gamma^{1-\lambda }M_{p}\left(f\right)+\gamma^{-\lambda }\;M_{q}\left(f\right)\right),$$

holds for all γ>0. Evaluating this inequality with

$$\gamma =\frac{\lambda \;M_{q}\left(f\right)}{\left(1-\lambda \right)M_{p}\left(f\right)},$$

leads to the stated result. □

The special case r=1/2 admits the simplified expression

(8) $$\psi_{1/2}\left(p,q\right)=\frac{\pi \lambda^{-\lambda }{\left(1-\lambda \right)}^{-(1-\lambda )}}{\left(q-p)\sin(\pi \lambda \right)},$$

where we have used Euler’s reflection formula for the beta function ([25], [Theorem 1.2.1]).

Next, we consider an extension of Proposition 2 for functions defined on $R^{n}$. Given any measurable subset *S* of $R^{n}$, we define

(9) $$\omega \left(S\right)=\mathrm{Vol}(B^{n}\cap \mathrm{cone}\left(S\right)),$$

where $B^{n}=\{u\in R^{n}:∥u∥\leq 1\}$ is the *n*-dimensional Euclidean ball of radius one and

$$\mathrm{cone}\left(S\right)=\left\{x\in R^{n}\;:\;tx\in S\;\mathrm{for}\;\mathrm{some}\;t>0\right\}.$$

The function ω(S) is proportional to the surface measure of the projection of *S* on the Euclidean sphere and satisfies

(10) $$\omega \left(S\right)\leq \omega \left(R^{n}\right)=\frac{\pi^{\frac{n}{2}}}{\Gamma (\frac{n}{2}+1)},$$

for all $S\subseteq R^{n}$. Note that $\omega (R_{+})=1$ and ω(R)=2.

**Proposition** **3.** *Let f be a nonnegative Lebesgue measurable function defined on a subset S of $R^{n}$. For any numbers p,q,r with 0<r<1 and p<1/r−1<q,*

$${∥f∥}_{r}\leq {\left[\omega \left(S\right)\;\psi_{r}\left(p,q\right)\right]}^{\frac{1-r}{r}}\;{\left[M_{np}\left(f\right)\right]}^{\lambda }{\left[M_{nq}\left(f\right)\right]}^{1-\lambda },$$

*where λ=(q+1−1/r)/(q−p) and $\psi_{r}\left(p,q\right)$ is given by Equation (7).*

**Proof.**  Let *f* be extended to $R^{n}$ using the rule f(x)=0 for all *x* outside of *S* and let $g:R_{+}\to R_{+}$ be defined according to

$$g\left(y\right)=\frac{1}{n}\int_{S^{n-1}}f\left(y^{1/n}u\right)\;d\sigma \left(u\right),$$

where $S^{n-1}=\{u\in R^{n}\;:\;∥u∥=1\}$ is the Euclidean sphere of radius one and σ(u) is the surface measure of the sphere. In the following, we will show that

(11) $$\begin{array}{cc} {∥f∥}_{r} & \leq {\left(\omega (S)\right)}^{\frac{1-r}{r}}{∥g∥}_{r} \end{array}$$

(12) $$\begin{array}{cc} M_{ns}\left(f\right) & =M_{s}\left(g\right). \end{array}$$

Then, the stated inequality then follows from applying Proposition 2 to the function *g*.

To prove Inequality (11), we begin with a transformation into polar coordinates:

(13) $${∥f∥}_{r}^{r}=\int_{0}^{\infty }\int_{S^{n-1}}{\left|f(tu)\right|}^{r}t^{n-1}\;d\sigma \left(u\right)\;dt.$$

Letting $\;\;1_{\mathrm{cone}(S)}\left(x\right)$ denote the indicator function of the set cone(S), the integral over the sphere can be bounded using:

(14) $$\begin{array}{cc} \int_{S^{n-1}}{\left|f(tu)\right|}^{r}\;d\sigma \left(u\right)\; & =\int_{S^{n-1}}\;\;1_{\mathrm{cone}(S)}\left(u\right)\;{\left|f(tu)\right|}^{r}\;d\sigma \left(u\right)\\ \; & \overset{\left(a\right)}{\leq }{\left(\int_{S^{n-1}}\;\;\;1_{\mathrm{cone}(S)}\left(u\right)\;d\sigma \left(u\right)\;\right)}^{\;1-r}{\left(\int_{S^{n-1}}\left|f(tu)\right|\;d\sigma \left(u\right)\;\right)}^{r}\\ \; & \overset{\left(b\right)}{=}n\;{\left(\omega (S)\right)}^{1-r}g^{r}\left(t^{n}\right), \end{array}$$

where: (a) follows from Hölder’s inequality with conjugate exponents $\frac{1}{1-r}$ and $\frac{1}{r}$, and (b) follows from the definition of *g* and the fact that

$$\begin{array}{cc} \omega (S) & =\int_{0}^{1}\int_{S^{n-1}}\;\;\;1_{\mathrm{cone}(S)}\left(u\right)\;t^{n-1}\;d\sigma \left(u\right)\;dt\\ \; & =\frac{1}{n}\int_{S^{n-1}}\;\;\;1_{\mathrm{cone}(S)}\left(u\right)\;d\sigma \left(u\right). \end{array}$$

Plugging Inequality (14) back into Equation (13) and then making the change of variable $t\to y^{\frac{1}{n}}$ yields

$${∥f∥}_{r}^{r}\leq n\;{\left(\omega (S)\right)}^{1-r}\int_{0}^{\infty }g^{r}\left(t^{n}\right)t^{n-1}\;dt={\left(\omega (S)\right)}^{1-r}{∥g∥}_{r}^{r}.$$

The proof of Equation (12) follows along similar lines. We have

$$\begin{array}{cc} M_{ns}\left(f\right)\; & \overset{\left(a\right)}{=}\int_{0}^{\infty }\int_{S^{n-1}}t^{ns}f\left(tu\right)\;t^{n-1}\;d\sigma \left(u\right)\;dt\\ \; & \overset{\left(b\right)}{=}\frac{1}{n}\int_{0}^{\infty }\int_{S^{n-1}}y^{s}f\left(y^{\frac{1}{n}}u\right)\;d\sigma \left(u\right)\;dy\\ \; & =M_{s}\left(g\right) \end{array}$$

where (a) follows from a transformation into polar coordinates and (b) follows from the change of variable $t\mapsto y^{\frac{1}{n}}$.

Having established Inequality (11) and Equation (12), an application of Proposition 2 completes the proof. □

## 3. Rényi Entropy Bounds

Let *X* be a random vector that has a density f(x) with respect to the Lebesgue measure on $R^{n}$. The differential Rényi entropy of order r∈(0,1)∪(1,∞) is defined according to [11]:

$$h_{r}\left(X\right)=\frac{1}{1-r}\log\left(\int_{R^{n}}f^{r}\left(x\right)\;dx\right).$$

Throughout this paper, it is assumed that the logarithm is defined with respect to the natural base and entropy is measured in nats. The Rényi entropy is continuous and nonincreasing in *r*. If the support set $S=\{x\in R^{n}:f\left(x\right)>0\}$ has finite measure, then the limit as *r* converges to zero is given by $h_{0}\left(X\right)=\log\mathrm{Vol}\left(S\right)$. If the support does not have finite measure, then $h_{r}\left(X\right)$ increases to infinity as *r* decreases to zero. The case r=1 is given by the Shannon differential entropy:

$$h_{1}\left(X\right)=-\int_{S}f\left(x\right)\log f\left(x\right)\;dx.$$

Given a random variable *X* that is not identical to zero and numbers p,q,r with 0<r<1 and p<1/r−1<q, we define the function

$$L_{r}\left(X;p,q\right)=\frac{r\lambda }{1-r}\log E\left[{\left|X\right|}^{p}\right]+\frac{r(1-\lambda )}{1-r}\log E\left[{\left|X\right|}^{q}\right],$$

where λ=(q+1−1/r)/(q−p).

The next result, which follows directly from Proposition 3, provides an upper bound on the Rényi entropy.

**Proposition** **4.** *Let X be a random vector with a density on $R^{n}$. For any numbers p,q,r with 0<r<1 and p<1/r−1<q, the Rényi entropy satisfies*

(15) $$h_{r}\left(X\right)\leq \log\omega \left(S\right)+\log\psi_{r}\left(p,q\right)+L_{r}{(∥X∥}^{n};p,q),$$

*where ω(S) is defined in Equation (9) and $\psi_{r}\left(p,q\right)$ is defined in Equation (7).*

**Proof.**  This result follows immediately from Proposition 3 and the definition of Rényi entropy. □

The relationship between Proposition 4 and previous results depends on whether the moment *p* is equal to zero:

- *One-moment inequalities:* If p=0, then there exists a distribution such that Inequality (15) holds with equality. This is because the zero-moment constraint ensures that the function that maximizes the Rényi entropy integrates to one. In this case, Proposition 4 is equivalent to previous results that focused on distributions that maximize Rényi entropy subject to a single moment constraint [12,13,15]. With some abuse of terminology, we refer to these bounds as one-moment inequalities. (A more accurate name would be two-moment inequalities under the constraint that one of the moments is the zeroth moment.)
- *Two-moment inequalities:* If p≠0, then the right-hand side of Inequality (15) corresponds to the Rényi entropy of a nonnegative function that might not integrate to one. Nevertheless, the expression provides an upper bound on the Rényi entropy for any density with the same moments. We refer to the bounds obtained using a general pair (p,q) as two-moment inequalities.

The contribution of two-moment inequalities is that they lead to tighter bounds. To quantify the tightness, we define $\Delta_{r}\left(X;p,q\right)$ to be the gap between the right-hand side and left-hand side of Inequality (15) corresponding to the pair (p,q)—that is,

$$\begin{array}{cc} \Delta_{r}\left(X;p,q\right) & =\log\omega \left(S\right)+\log\psi_{r}\left(p,q\right)+L_{r}{(∥X∥}^{n};p,q)-h_{r}\left(X\right). \end{array}$$

The gaps corresponding to the optimal two-moment and one-moment inequalities are defined according to

$$\begin{array}{cc} \Delta_{r}\left(X\right) & =\underset{p,q}{\inf}\Delta_{r}\left(X;p,q\right)\\ {\tilde{\Delta }}_{r}\left(X\right) & =\underset{q}{\inf}\Delta_{r}\left(X;0,q\right). \end{array}$$

### 3.1. Some Consequences of These Bounds

By Lyapunov’s inequality, the mapping $s\mapsto \frac{1}{s}\log E\left[{\left|X\right|}^{s}\right]$ is nondecreasing on [0,∞), and thus

(16) $$L_{r}\left(X;p,q\right)\leq L_{r}\left(X;0,q\right)=\frac{1}{q}\log E\left[{\left|X\right|}^{q}\right],\;p\geq 0.$$

In other words, the case p=0 provides an upper bound on $L_{r}\left(X;p,q\right)$ for nonnegative *p*. Alternatively, we also have the lower bound

(17) $$L_{r}\left(X;p,q\right)\geq \frac{r}{1-r}\log E\left[{\left|X\right|}^{\frac{1-r}{r}}\right],$$

which follows from the convexity of $\log E\left[{\left|X\right|}^{s}\right]$.

A useful property of $L_{r}\left(X;p,q\right)$ is that it is additive with respect to the product of independent random variables. Specifically, if *X* and *Y* are independent, then

(18) $$\begin{array}{c} L_{r}\left(XY;p,q\right)=L_{r}\left(X;p,q\right)+L_{r}\left(Y;p,q\right). \end{array}$$

One consequence is that multiplication by a bounded random variable cannot increase the Rényi entropy by an amount that exceeds the gap of the two-moment inequality with nonnegative moments.

**Proposition** **5.** *Let Y be a random vector on $R^{n}$ with finite Rényi entropy of order 0<r<1, and let X be an independent random variable that satisfies 0<X≤t. Then,*

$$h_{r}\left(XY\right)\leq h_{r}\left(tY\right)+\Delta_{r}\left(Y;p,q\right),$$

*for all 0<p<1/r−1<q.*

**Proof.**  Let Z=XY and let $S_{Z}$ and $S_{Y}$ denote the support sets of *Z* and *Y*, respectively. The assumption that *X* is nonnegative means that $\mathrm{cone}\left(S_{Z}\right)=\mathrm{cone}\left(S_{Y}\right)$. We have

$$\begin{array}{cc} h_{r}\left(Z\right) & \overset{\left(a\right)}{\leq }\log\omega \left(S_{Z}\right)+\log\psi_{r}\left(p,q\right)+L_{r}{(∥Z∥}^{n};p,q)\\ \; & \overset{\left(b\right)}{=}h_{r}\left(Y\right)+L_{r}{\left(|X\right|}^{n};p;q)+\Delta_{r}\left(Y;p,q\right)\\ \; & \overset{\left(c\right)}{\leq }h_{r}\left(Y\right)+n\log t+\Delta_{r}\left(Y;p,q\right), \end{array}$$

where (a) follows from Proposition 4, (b) follows from Equation (18) and the definition of $\Delta_{r}\left(Y;p,q\right)$, and (c) follows from Inequality (16) and the assumption |X|≤t. Finally, recalling that $h_{r}\left(tY\right)=h_{r}\left(Y\right)+n\log t$ completes the proof. □

### 3.2. Example with Log-Normal Distribution

If $W∼N(\mu ,\sigma^{2})$, then the random variable X=exp(W) has a log-normal distribution with parameters $\left(\mu ,\sigma^{2}\right)$. The Rényi entropy is given by

$$h_{r}\left(X\right)=\mu +\frac{1}{2}\left(\frac{1-r}{r}\right)\sigma^{2}+\frac{1}{2}\log\left(2\pi r^{\frac{1}{r-1}}\sigma^{2}\right),$$

and the logarithm of the *s*th moment is given by

$$\log E\left[{\left|X\right|}^{s}\right]=\mu s\;+\frac{1}{2}\sigma^{2}\;s^{2}.$$

With a bit of work, it can be shown that the gap of the optimal two-moment inequality does not depend on the parameters $\left(\mu ,\sigma^{2}\right)$ and is given by

(19) $$\begin{array}{cc} \Delta_{r}\left(X\right) & =\log\left(\tilde{B}\left(\frac{r}{2(1-r)},\frac{r}{2(1-r)}\right)\sqrt{\frac{r}{4(1-r)}}\;\right)+\frac{1}{2}-\frac{1}{2}\log\left(2\pi r^{\frac{1}{r-1}}\right). \end{array}$$

The details of this derivation are given in Appendix B.1. Meanwhile, the gap of the optimal one-moment inequality is given by

(20) $$\begin{array}{cc} {\tilde{\Delta }}_{r}\left(X\right) & =\underset{q}{\inf}\left[\log\left(\tilde{B}\left(\frac{r}{1-r}-\frac{1}{q},\frac{1}{q}\right)\frac{1}{q}\right)+\frac{1}{2}q\sigma^{2}\right]-\frac{1}{2}\left(\frac{1-r}{r}\right)\sigma^{2}-\frac{1}{2}\log\left(2\pi r^{\frac{1}{r-1}}\sigma^{2}\right). \end{array}$$

**Figure 1:**
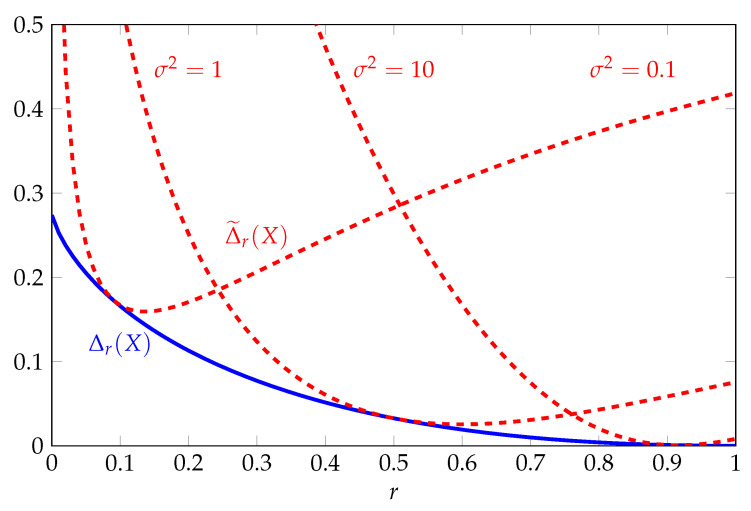
Comparison of upper bounds on Rényi entropy in nats for the log-normal distribution as a function of the order *r* for various $\sigma^{2}$.

The functions $\Delta_{r}\left(X\right)$ and ${\tilde{\Delta }}_{r}\left(X\right)$ are illustrated in Figure 1 as a function of *r* for various $\sigma^{2}$. The function $\Delta_{r}\left(X\right)$ is bounded uniformly with respect to *r* and converges to zero as *r* increases to one. The tightness of the two-moment inequality in this regime follows from the fact that the log-normal distribution maximizes Shannon entropy subject to a constraint on $E\left[\log X\right]$. By contrast, the function ${\tilde{\Delta }}_{r}\left(X\right)$ varies with the parameter $\sigma^{2}$. For any fixed r∈(0,1), it can be shown that ${\tilde{\Delta }}_{r}\left(X\right)$ increases to infinity if $\sigma^{2}$ converges to zero or infinity.

### 3.3. Example with Multivariate Gaussian Distribution

Next, we consider the case where $Y∼N(0,I_{n})$ is an *n*-dimensional Gaussian vector with mean zero and identity covariance. The Rényi entropy is given by

$$h_{r}\left(Y\right)=\frac{n}{2}\log\left(2\pi r^{\frac{1}{r-1}}\right),$$

and the *s*th moment of the magnitude ∥Y∥ is given by

$$E\left[{∥Y∥}^{s}\right]=\frac{2^{\frac{s}{2}}\Gamma \left(\frac{n+s}{2}\right)}{\Gamma (\frac{n}{2})}.$$

The next result shows that as the dimension *n* increases, the gap of the optimal two-moment inequality converges to the gap for the log-normal distribution. Moreover, for each r∈(0,1), the following choice of moments is optimal in the large-*n* limit:

(21) $$\begin{array}{cc} p_{n} & =\frac{1-r}{r}-\frac{1}{r}\sqrt{\frac{2(1-r)}{n+1}},\;q_{n}=\frac{1-r}{r}+\frac{1}{r}\sqrt{\frac{2(1-r)}{n+1}}. \end{array}$$

The proof is given in Appendix B.3.

**Proposition** **6.** *If $Y∼N(0,I_{n})$, then, for each r∈(0,1),*

$$\lim_{n\to \infty }\Delta_{r}\left(Y\right)=\lim_{n\to \infty }\Delta_{r}\left(Y;p_{n},q_{n}\right)=\Delta_{r}\left(X\right),$$

*where X has a log-normal distribution and $\left(p_{n},q_{n}\right)$ are given by (21).*

**Figure 2:**
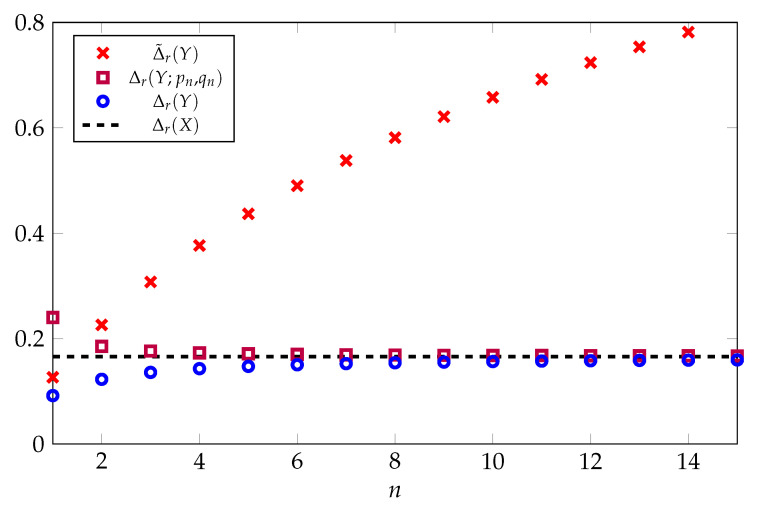
Comparison of upper bounds on Rényi entropy in nats for the multivariate Gaussian distribution $N(0,I_{n})$ as a function of the dimension *n* with r=0.1. The solid black line is the gap of the optimal two-moment inequality for the log-normal distribution.

Figure 2 provides a comparison of $\Delta_{r}\left(Y\right)$, $\Delta_{r}\left(Y;p_{n},q_{n}\right)$, and ${\tilde{\Delta }}_{r}\left(Y\right)$ as a function of *n* for r=0.1. Here, we see that both $\Delta_{r}\left(Y\right)$ and $\Delta_{r}\left(Y;p_{n},q_{n}\right)$ converge rapidly to the asymptotic limit given by the gap of the log-normal distribution. By contrast, the gap of the optimal one-moment inequality ${\tilde{\Delta }}_{r}\left(Y\right)$ increases without bound.

### 3.4. Inequalities for Differential Entropy

Proposition 4 can also be used to recover some known inequalities for differential entropy by considering the limiting behavior as *r* converges to one. For example, it is well known that the differential entropy of an *n*-dimensional random vector *X* with finite second moment satisfies

(22) $$\begin{array}{c} h\left(X\right)\leq \frac{1}{2}\log\left(2\pi e\;E\left[\frac{1}{n}{∥X∥}^{2}\right]\right), \end{array}$$

with equality if and only if the entries of *X* are i.i.d. zero-mean Gaussian. A generalization of this result in terms of an arbitrary positive moment is given by

(23) $$\begin{array}{cc} h(X) & \leq \log\frac{\Gamma \left(\frac{n}{s}+1\right)}{\Gamma \left(\frac{n}{2}+1\right)}+\frac{n}{2}\log\pi +\frac{n}{s}\log\left(es\;E\left[\frac{1}{n}{∥X∥}^{s}\right]\right), \end{array}$$

for all s>0. Note that Inequality (22) corresponds to the case s=2.

Inequality (23) can be proved as an immediate consequence of Proposition 4 and the fact that $h_{r}\left(X\right)$ is nonincreasing in *r*. Using properties of the beta function given in Appendix A, it is straightforward to verify that

$$\lim_{r\to 1}\psi_{r}\left(0,q\right)={\left(e\;q\right)}^{\frac{1}{q}}\Gamma \left(\frac{1}{q}+1\right),\;\mathrm{for}\;\mathrm{all}\;q>0.$$

Combining this result with Proposition 4 and Inequality (16) leads to

$$\begin{array}{cc} h(X) & \leq \log\omega \left(S\right)+\log\Gamma \left(\frac{1}{q}+1\right)+\frac{1}{q}\log\left(eqE\left[{∥X∥}^{nq}\right]\right). \end{array}$$

Using Inequality (10) and making the substitution s=nq leads to Inequality (23).

Another example follows from the fact that the log-normal distribution maximizes the differential entropy of a positive random variable *X* subject to constraints on the mean and variance of log(X), and hence

(24) $$\begin{array}{cc} h(X) & \leq E\left[\log(X)\right]+\frac{1}{2}\log\left(2\pi e\mathrm{Var}(\log(X))\right), \end{array}$$

with equality if and only if *X* is log-normal. In Appendix B.4, it is shown how this inequality can be proved using our two-moment inequalities by studying the behavior as both *p* and *q* converge to zero as *r* increases to one.

## 4. Bounds on Mutual Information

### 4.1. Relative Entropy and Chi-Squared Divergence

Let *P* and *Q* be distributions defined on a common probability space that have densities *p* and *q* with respect to a dominating measure μ. The relative entropy (or Kullback–Leibler divergence) is defined according to

$$D\left(P\;∥\;Q\right)=\int p\log\left(\frac{p}{q}\right)d\mu ,$$

and the chi-squared divergence is defined as

$$\chi^{2}\left(P\;∥\;Q\right)=\int \frac{{\left(p-q\right)}^{2}}{q}d\mu .$$

Both of these divergences can be seen as special cases of the general class of *f*-divergence measures and there exists a rich literature on comparisons between different divergences [8,26,27,28,29,30,31,32]. The chi-squared divergence can also be viewed as the squared $L_{2}$ distance between $p/\sqrt{q}$ and $\sqrt{q}$. The chi-square can also be interpreted as the first non-zero term in the power series expansion of the relative entropy ([26], [Lemma 4]). More generally, the chi-squared divergence provides an upper bound on the relative entropy via

(25) $$\begin{array}{cc} D\left(P\;∥\;Q\right) & \leq \log(1+\chi^{2}\left(P∥Q\right)). \end{array}$$

The proof of this inequality follows straightforwardly from Jensen’s inequality and the concavity of the logarithm; see [27,31,32] for further refinements.

Given a random pair (X,Y), the mutual information between *X* and *Y* is defined according to

$$I\left(X;Y\right)=D\left(P_{X,Y}\;∥\;P_{X}P_{Y}\right).$$

From Inequality (25), we see that the mutual information can always be upper bounded using

(26) $$\begin{array}{c} I\left(X;Y\right)\leq \log(1+\chi^{2}\left(P_{X,Y}∥P_{X}P_{Y}\right)). \end{array}$$

The next section provides bounds on the mutual information that can improve upon this inequality.

### 4.2. Mutual Information and Variance of Conditional Density

Let (X,Y) be a random pair such that the conditional distribution of *Y* given *X* has a density $f_{Y|X}\left(y|x\right)$ with respect to the Lebesgue measure on $R^{n}$. Note that the marginal density of *Y* is given by $f_{Y}\left(y\right)=E\left[f_{Y|X}\left(y|X\right)\right]$. To simplify notation, we will write f(y|x) and f(y) where the subscripts are implicit. The support set of *Y* is denoted by $S_{Y}$.

The measure of the dependence between *X* and *Y* that is used in our bounds can be understood in terms of the variance of the conditional density. For each *y*, the conditional density f(y|X) evaluated with a random realization of *X* is a random variable. The variance of this random variable is given by

(27) $$\begin{array}{c} \mathrm{Var}\left(f\left(y|X\right)\right)=E\left[{\left(f(y|X)-f(y)\right)}^{2}\right], \end{array}$$

where we have used the fact that the marginal density f(y) is the expectation of f(y|X). The *s*th moment of the variance of the conditional density is defined according to

(28) $$\begin{array}{c} V_{s}\left(Y|X\right)=\int_{S_{Y}}{∥y∥}^{s}\mathrm{Var}\left(f\left(y|X\right)\right)\;dy. \end{array}$$

The variance moment $V_{s}\left(Y|X\right)$ is nonnegative and equal to zero if and only if *X* and *Y* are independent.

The function κ(t) is defined according to

(29) $$\begin{array}{c} \kappa \left(t\right)=\underset{u\in (0,\infty )}{\sup}\frac{\log(1+u)}{u^{t}},\;t\in \left(0,1\right]. \end{array}$$

**Figure 3:**
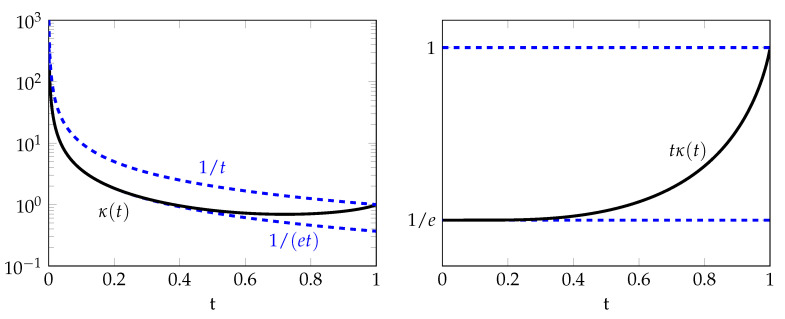
Graphs of κ(t) and tκ(t) as a function of *t*.

The proof of the following result is given in Appendix C. The behavior of κ(t) is illustrated in Figure 3.

**Proposition** **7.** *The function κ(t) defined in Equation (29) can be expressed as*

$$\kappa \left(t\right)=\frac{\log(1+u)}{u^{t}},\;t\in \left(0,1\right]$$

*where*

$$u=\exp\left(W\left(-\frac{1}{t}\exp\left(-\frac{1}{t}\right)\right)+\frac{1}{t}\right)-1,$$

*and W(·) denotes Lambert’s W- function, i.e., W(z) is the unique solution to the equation z=wexp(w) on the interval [−1,∞). Furthermore, the function g(t)=tκ(t) is strictly increasing on (0,1] with $\lim_{t\to 0}g\left(t\right)=1/e$ and g(1)=1, and thus*

$$\frac{1}{et}\leq \kappa \left(t\right)\leq \frac{1}{t},\;t\in \left(0,1\right],$$

*where the lower bound 1/(et) is tight for small values of t∈(0,1) and the upper bound 1/t is tight for values of t close to 1.*

We are now ready to give the main results of this section, which are bounds on the mutual information. We begin with a general upper bound in terms of the variance of the conditional density.

**Proposition** **8.** *For any 0<t≤1, the mutual information satisfies*

$$I\left(X;Y\right)\leq \kappa \left(t\right)\int_{S_{Y}}{\left[f(y)\right]}^{1-2t}\;{\left[\mathrm{Var}(f(y|X))\right]}^{t}\;dy.$$

**Proof.**  We use the following series of inequalities:

$$\begin{array}{cc} I(X;Y) & \overset{\left(a\right)}{=}\int f\left(y\right)\;D\left(P_{X|Y=y}\;∥\;P_{X}\right)\;dy\\ \; & \overset{\left(b\right)}{\leq }\int f\left(y\right)\;\log\left(1+\chi^{2}\left(P_{X|Y=y}∥P_{X}\right)\right)\;dy\\ \; & \overset{\left(c\right)}{=}\int f\left(y\right)\;\log\left(1+\frac{\mathrm{Var}(f(y|X))}{f^{2}\left(y\right)}\right)\;dy\\ \; & \overset{\left(d\right)}{\leq }\kappa \left(t\right)\int f\left(y\right){\left(\frac{\mathrm{Var}(f(y|X))}{f^{2}\left(y\right)}\right)}^{t}\;dy, \end{array}$$

where (a) follows from the definition of mutual information, (b) follows from Inequality (25), and (c) follows from Bayes’ rule, which allows us to write the chi-square in terms of the variance of the conditional density:

$$\chi^{2}\left(P_{X|Y=y}∥P_{X}\right)=E\left[{\left(\;\frac{f(y|X)}{f(y)}-1\right)}^{2}\right]=\frac{\mathrm{Var}(f(y|X))}{f^{2}\left(y\right)}.$$

Inequality (d) follows from the nonnegativity of the variance and the definition of κ(t). □

Evaluating Proposition 8 with t=1 recovers the well-known inequality $I\left(X;Y\right)\leq \chi^{2}\left(P_{X,Y}∥P_{X}P_{Y}\right)$. The next two results follow from the cases $0<t<\frac{1}{2}$ and $t=\frac{1}{2}$, respectively.

**Proposition** **9.** *For any 0<r<1, the mutual information satisfies*

$$I\left(X;Y\right)\leq \kappa \left(t\right){\left(e^{h_{r}\left(Y\right)}\;V_{0}\left(Y|X\right)\right)}^{t},$$

*where t=(1−r)/(2−r).*

**Proof.**  Starting with Proposition 8 and applying Hölder’s inequality with conjugate exponents 1/(1−t) and 1/t leads to

$$\begin{array}{cc} I(X;Y)\; & \leq \kappa \left(t\right){\left(\int f^{r}\left(y\right)\;dy\right)}^{1-t}{\left(\int \mathrm{Var}(f(y|X))\;dy\right)}^{t}=\kappa \left(t\right)\;e^{t\;h_{r}\left(Y\right)}V_{0}^{t}\left(Y|X\right), \end{array}$$

where we have used the fact that r=(1−2t)/(1−t). □

**Proposition** **10.** *For any p<1<q, the mutual information satisfies*

$$I\left(X;Y\right)\leq C\left(\lambda \right)\;\sqrt{\frac{\omega \left(S_{Y}\right)V_{np}^{\lambda }\left(Y|X\right)V_{nq}^{1-\lambda }\left(Y|X\right)}{\left(q-p\right)}},$$

*where λ=(q−1)/(q−p) and*

$$C\left(\lambda \right)=\kappa \left(\frac{1}{2}\right)\sqrt{\frac{\pi \lambda^{-\lambda }{\left(1-\lambda \right)}^{-(1-\lambda )}}{\sin(\pi \lambda )}},$$

*with $\kappa (\frac{1}{2})=0.80477\cdots$.*

**Proof.**  Evaluating Proposition 8 with t=1/2 gives

$$I\left(X;Y\right)\leq \kappa \left(\frac{1}{2}\right)\int_{S_{Y}}\sqrt{\mathrm{Var}(f(y|X))}\;dy.$$

Evaluating Proposition 3 with $r=\frac{1}{2}$ leads to

$$\begin{array}{cc} {\left(\int_{S_{Y}}\sqrt{\mathrm{Var}(f(y|X))}\;dy\right)}^{2}\; & \leq \omega \left(S_{Y}\right)\;\psi_{1/2}\left(p,q\right)V_{np}^{\lambda }\left(Y|X\right)V_{nq}^{1-\lambda }\left(Y|X\right). \end{array}$$

Combining these inequalities with the expression for $\psi_{1/2}\left(p,q\right)$ given in Equation (8) completes the proof. □

The contribution of Propositions 9 and 10 is that they provide bounds on the mutual information in terms of quantities that can be easy to characterize. One application of these bounds is to establish conditions under which the mutual information corresponding to a sequence of random pairs $\left(X_{k},Y_{k}\right)$ converges to zero. In this case, Proposition 9 provides a sufficient condition in terms of the Rényi entropy of $Y_{n}$ and the function $V_{0}\left(Y_{n}|X_{n}\right)$, while Proposition 10 provides a sufficient condition in terms of $V_{s}\left(Y_{n}|X_{n}\right)$ evaluated with two difference values of *s*. These conditions are summarized in the following result.

**Proposition** **11.** *Let $\left(X_{k},Y_{k}\right)$ be a sequence of random pairs such that the conditional distribution of $Y_{k}$ given $X_{k}$ has a density on $R^{n}$. The following are sufficient conditions under which the mutual information of $I(X_{k};Y_{k})$ converges to zero as k increases to infinity:*

1. *There exists 0<r<1 such that*
  $$\begin{array}{cc} \lim_{k\to \infty }e^{h_{r}\left(Y_{k}\right)}V_{0}\left(Y_{k}|X_{k}\right) & =0. \end{array}$$
2. *There exists p<1<q such that*
  $$\begin{array}{c} \lim_{k\to \infty }V_{np}^{q-1}\left(Y_{k}|X_{k}\right)V_{nq}^{1-p}\left(Y_{k}|X_{k}\right)=0. \end{array}$$

### 4.3. Properties of the Bounds

The variance moment $V_{s}\left(Y|X\right)$ has a number of interesting properties. The variance of the conditional density can be expressed in terms of an expectation with respect to two independent random variables $X_{1}$ and $X_{2}$ with the same distribution as *X* via the decomposition:

$$\mathrm{Var}\left(f\left(y|X\right)\right)=E\left[f\left(y|X\right)f\left(y|X\right)-f\left(y|X_{1}\right)f\left(y|X_{2}\right)\right].$$

Consequently, by swapping the order of the integration and expectation, we obtain

(30) $$V_{s}\left(Y|X\right)=E\left[K_{s}\left(X,X\right)-K_{s}\left(X_{1},X_{2}\right)\right],$$

where

$$K_{s}\left(x_{1},x_{2}\right)=\int {∥y∥}^{s}f\left(y|x_{1}\right)f\left(y|x_{2}\right)\;dy.$$

The function $K_{s}\left(x_{1},x_{2}\right)$ is a positive definite kernel that does not depend on the distribution of *X*. For s=0, this kernel has been studied previously in the machine learning literature [33], where it is referred to as the expected likelihood kernel.

The variance of the conditional density also satisfies a data processing inequality. Suppose that U→X→Y forms a Markov chain. Then, the square of the conditional density of *Y* given *U* can be expressed as

$$f_{Y|U}^{2}\left(y|u\right)=E\left[f_{Y|X}\left(y|X_{1}^{\prime }\right)f_{Y|X}\left(y|X_{2}^{\prime }\right)|U=u\right],$$

where $\left(U,X_{1}^{\prime },X_{2}^{\prime }\right)∼P_{U}P_{X_{1}|U}P_{X_{2}|U}$. Combining this expression with Equation (30) yields

(31) $$\begin{array}{c} V_{s}\left(Y|U\right)=E\left[K_{s}\left(X_{1}^{\prime },X_{2}^{\prime }\right)-K_{s}\left(X_{1},X_{2}\right)\right], \end{array}$$

where we recall that $\left(X_{1},X_{2}\right)$ are independent copies of *X*

Finally, it is easy to verify that the function $V_{s}\left(Y\right)$ satisfies

$$V_{s}\left(aY|X\right)={\left|a\right|}^{s-n}V_{s}\left(Y|X\right),\;\;\mathrm{for}\;\mathrm{all}\;a\neq 0.$$

Using this scaling relationship, we see that the sufficient conditions in Proposition 11 are invariant to scaling of *Y*.

### 4.4. Example with Additive Gaussian Noise

We now provide a specific example of our bounds on the mutual information. Let $X\in R^{n}$ be a random vector with distribution $P_{X}$ and let *Y* be the output of a Gaussian noise channel

(32) Y=X+W,

where $W∼N(0,I_{n})$ is independent of *X*. If ∥X∥ has finite second moment, then the mutual information satisfies

(33) $$I\left(X;Y\right)\leq \frac{n}{2}\log\left(1+\frac{1}{n}E\left[{∥X∥}^{2}\right]\right),$$

where equality is attained if and only if *X* has zero-mean isotropic Gaussian distribution. This inequality follows straightforwardly from the fact that the Gaussian distribution maximizes differential entropy subject to a second moment constraint [11]. One of the limitations of this bound is that it can be loose when the second moment is dominated by events that have small probability. In fact, it is easy to construct examples for which ∥X∥ does not have a finite second moment, and yet I(X;Y) is arbitrarily close to zero.

Our results provide bounds on I(X;Y) that are less sensitive to the effects of rare events. Let $\phi_{n}\left(x\right)={\left(2\pi \right)}^{-n/2}{\exp(-∥x∥}^{2}/2)$ denote the density of the standard Gaussian distribution on $R^{n}$. The product of the conditional densities can be factored according to

$$\begin{array}{cc} f\left(y|x_{1}\right)f\left(y|x_{2}\right)\; & =\phi_{2n}\left(\left[\begin{array}{c} y-x_{1}\\ y-x_{2} \end{array}\right]\right)=\phi_{2n}\left(\left[\begin{array}{c} \sqrt{2}y-\left(x_{1}+x_{2}\right)/\sqrt{2}\\ \left(x_{1}-x_{2}\right)/\sqrt{2} \end{array}\right]\right)\\ \; & =\phi_{n}\left(\sqrt{2}\;y-\frac{x_{1}+x_{2}}{\sqrt{2}}\right)\phi_{n}\left(\frac{x_{1}-x_{2}}{\sqrt{2}}\right), \end{array}$$

where the second step follows because $\phi_{2n}\left(\cdot \right)$ is invariant to orthogonal transformations. Integrating with respect to *y* leads to

$$\begin{array}{cc} K_{s}\left(x_{1},x_{2}\right) & =2^{-\frac{n+s}{2}}E\left[{∥W+\frac{x_{1}+x_{2}}{\sqrt{2}}∥}^{s}\right]\phi_{n}\left(\frac{x_{1}-x_{2}}{\sqrt{2}}\right), \end{array}$$

where we recall that $W∼N(0,I_{n})$. For the case s=0, we see that $K_{0}\left(x_{1},x_{2}\right)$ is a Gaussian kernel, thus

(34) $$\begin{array}{c} V_{0}\left(Y|X\right)={\left(4\pi \right)}^{-\frac{n}{2}}\left[1-E\left[e^{-\frac{1}{4}{∥X_{1}-X_{2}∥}^{2}}\right]\right]. \end{array}$$

A useful property of $V_{0}\left(Y|X\right)$ is that the conditions under which it converges to zero are weaker than the conditions needed for other measures of dependence. Observe that the expectation in Equation (34) is bounded uniformly with respect to $\left(X_{1},X_{2}\right)$. In particular, for every ϵ>0 and x∈R, we have

$$\begin{array}{cc} 1-E\left[e^{-\frac{1}{4}{\left(X_{1}-X_{2}\right)}^{2}}\right]\; & \leq \epsilon^{2}+2P\left[|X-x|\geq \epsilon \right], \end{array}$$

where we have used the inequality $1-e^{-x}\leq x$ and the fact that $P\left[|X_{1}-X_{2}|\geq 2\epsilon \right]\leq 2P\left[|X-x|\geq \epsilon \right]$. Consequently, $V_{0}\left(Y|X\right)$ converges to zero whenever *X* converges to a constant value *x* in probability.

To study some further properties of these bounds, we now focus on the case where *X* is a Gaussian scalar mixture generated according to

(35) $$X=A\sqrt{U},\;A∼N\left(0,1\right),\;U\geq 0,$$

with *A* and *U* independent. In this case, the expectations with respect to the kernel $K_{s}\left(x_{1},x_{2}\right)$ can be computed explicitly, leading to

(36) $$\begin{array}{c} V_{s}\left(Y|X\right)=\frac{\Gamma (\frac{1+s}{2})}{2\pi }E\left[{\left(1+2U\right)}^{\frac{s}{2}}-\frac{{\left(1+U_{1}\right)}^{\frac{s}{2}}{\left(1+U_{2}\right)}^{\frac{s}{2}}}{{\left(1+\frac{1}{2}\left(U_{1}+U_{2}\right)\right)}^{\frac{s+1}{2}}}\right], \end{array}$$

where $\left(U_{1},U_{2}\right)$ are independent copies of *U*. It can be shown that this expression depends primarily on the magnitude of *U*. This is not surprising given that *X* converges to a constant if and only if *U* converges to zero.

Our results can also be used to bound the mutual information I(U;Y) by noting that U→X→Y forms a Markov chain, and taking advantage of the characterization provided in Equation (31). Letting $X_{1}^{\prime }=A_{1}\sqrt{U}$ and $X_{2}^{\prime }=A_{2}\sqrt{U}$ with $\left(A_{1},A_{2},U\right)$ be mutually independent leads to

(37) $$\begin{array}{c} V_{s}\left(Y|U\right)=\frac{\Gamma (\frac{1+s}{2})}{2\pi }E\left[{\left(1+U\right)}^{\frac{s-1}{2}}-\frac{{\left(1+U_{1}\right)}^{\frac{s}{2}}{\left(1+U_{2}\right)}^{\frac{s}{2}}}{{\left(1+\frac{1}{2}\left(U_{1}+U_{2}\right)\right)}^{\frac{s+1}{2}}}\right], \end{array}$$

In this case, $V_{s}\left(Y|U\right)$ is a measure of the variation in *U*. To study its behavior, we consider the simple upper bound

(38) $$\begin{array}{c} V_{s}\left(Y|U\right)\leq \frac{\Gamma (\frac{1+s}{2})}{2\pi }P\left[U_{1}\neq U_{2}\right]E\left[{\left(1+U\right)}^{\frac{s-1}{2}}\right], \end{array}$$

which follows from noting that the term inside the expectation in Equation (37) is zero on the event $U_{1}=U_{2}$. This bound shows that if s≤1 then $V_{s}\left(Y|U\right)$ is bounded uniformly with respect to distributions on *U*, and if s>1, then $V_{s}\left(Y|U\right)$ is bounded in terms of the $\left(\frac{s-1}{2}\right)$th moment of *U*.

**Figure 4:**
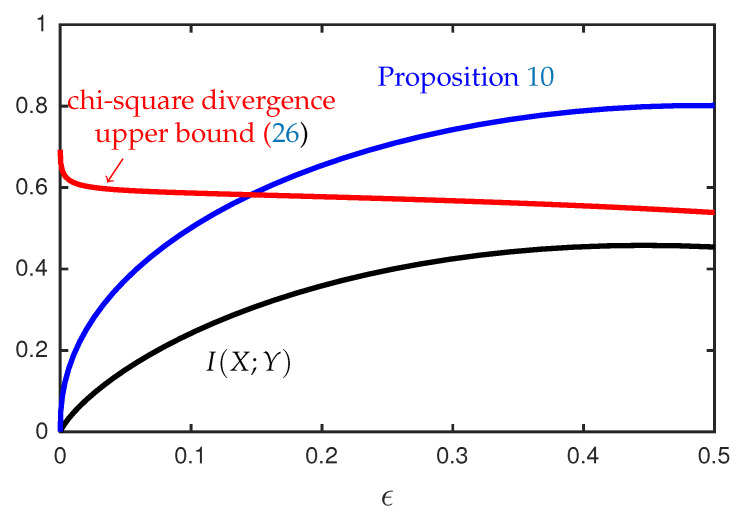
Bounds on the mutual information I(U;Y) in nats when $U∼\left(1-\epsilon \right)\delta_{1}+\epsilon \delta_{a(\epsilon )}$, with $a\left(\epsilon \right)=1+1/\sqrt{\epsilon }$, and *X* and *Y* are generated according to Equations (32) and (35). The bound from Proposition 10 is evaluated with p=0 and q=2.

In conjunction with Propositions 9 and 10, the function $V_{s}\left(Y|U\right)$ provides bounds on the mutual information I(U;Y) that can be expressed in terms of simple expectations involving two independent copies of *U*. Figure 4 provides an illustration of the upper bound in Proposition 10 for the case where *U* is a discrete random variable supported on two points, and *X* and *Y* are generated according to Equations (32) and (35). This example shows that there exist sequences of distributions for which our upper bounds on the mutual information converge to zero while the chi-squared divergence between $P_{XY}$ and $P_{X}P_{Y}$ is bounded away from zero.

## 5. Conclusions

This paper provides bounds on Rényi entropy and mutual information that are based on a relatively simple two-moment inequality. Extensions to inequalities with more moments are worth exploring. Another potential application is to provide a refined characterization of the “all-or-nothing” behavior seen in a sparse linear regression problem [34,35], where the current methods of analysis depend on a complicated conditional second moment method.

## Appendix A. The Gamma and Beta Functions

This section reviews some properties of the gamma and beta functions. For x>0, the gamma function is defined according to $\Gamma \left(x\right)=\int_{0}^{\infty }t^{x-1}e^{-t}\;dt.$ Binet’s formula for the logarithm for the gamma function ([25], [Theorem 1.6.3]) gives

(A1) $$\begin{array}{c} \log\Gamma \left(x\right)=\left(x-\frac{1}{2}\right)\log x-x+\frac{1}{2}\log\left(2\pi \right)+\theta \left(x\right), \end{array}$$

where the remainder term θ(x) is convex and nonincreasing with $\lim_{x\to 0}\theta \left(x\right)=\infty$ and $\lim_{x\to \infty }\theta \left(x\right)=0$. Euler’s reflection formula ([25], [Theorem 1.2.1]) gives

(A2) $$\begin{array}{c} \Gamma \left(x\right)\Gamma \left(1-x\right)=\frac{\pi }{\sin(\pi x)},\;0<x<1. \end{array}$$

For x,y>0, the beta function can be expressed as follows

(A3) $$\begin{array}{cc} B(x,y) & =\frac{\Gamma (x)\Gamma (y)}{\Gamma (x+y)}=\int_{0}^{1}t^{x-1}{\left(1-t\right)}^{y-1}\;dt=\int_{0}^{\infty }\frac{u^{a-1}}{{\left(1+u\right)}^{a+b}}\;du, \end{array}$$

where the second integral expression follows from the change of variables t↦u/(1+u). Recall that $\tilde{B}\left(x,y\right)=B\left(x,y\right){\left(x+y\right)}^{x+y}x^{-x}y^{-y}$. Using Equation (A1) leads to

(A4) $$\begin{array}{c} \log\left(\tilde{B}\left(x,y\right)\sqrt{\frac{x\;y}{2\pi (x\;+\;y)}}\right)=\theta \left(x\right)+\theta \left(y\right)-\theta \left(x+y\right). \end{array}$$

It can also be shown that ([36], [Equation (2), pg. 2])

(A5) $$\begin{array}{c} \tilde{B}\left(x,y\right)\geq \frac{x+y}{xy}. \end{array}$$

## Appendix B. Details for Rényi Entropy Examples

This appendix studies properties of the two-moment inequalities for Rényi entropy described in Section 3.

### Appendix B.1. Log-Normal Distribution

Let *X* be a log-normal random variable with parameters $\left(\mu ,\sigma^{2}\right)$ and consider the parametrization

$$\begin{array}{cc} p & =\frac{1-r}{r}-\left(1-\lambda \right)\sqrt{\frac{(1-r)\;u}{r\lambda (1-\lambda )}}\\ q & =\frac{1-r}{r}+\lambda \;\sqrt{\frac{(1-r)\;u}{r\lambda (1-\lambda )}}. \end{array}$$

where λ∈(0,1) and u∈(0,∞). Then, we have

$$\begin{array}{cc} \psi_{r}\left(p,q\right) & =\tilde{B}\left(\frac{r\lambda }{1-r},\frac{r(1-\lambda )}{1-r}\right)\sqrt{\frac{r\lambda (1-\lambda )}{(1-r)\;u}}\\ L_{r}\left(X;p,q\right) & =\mu +\frac{1}{2}\left(\frac{1-r}{r}\right)\sigma^{2}+\frac{1}{2}u\sigma^{2}. \end{array}$$

Combining these expressions with Equation (A4) leads to

(A6) $$\begin{array}{cc} \Delta_{r}\left(X;p,q\right)\; & =\theta \left(\frac{r\lambda }{1\;-\;r}\right)+\theta \left(\frac{r(1\;-\;\lambda )}{1-r}\right)-\theta \left(\frac{r}{1\;-\;r}\right)+\frac{1}{2}u\sigma^{2}-\frac{1}{2}\log\left(u\sigma^{2}\right)-\frac{1}{2}\log\left(r^{\frac{1}{r-1}}\right). \end{array}$$

We now characterize the minimum with respect to the parameters (λ,u). Note that the mapping $\lambda \mapsto \theta \left(\frac{r\lambda }{1-r}\right)+\theta \left(\frac{r(1-\lambda )}{1-r}\right)$ is convex and symmetric about the point λ=1/2. Therefore, the minimum with respect to λ is attained at λ=1/2. Meanwhile, mapping $u\mapsto u\sigma^{2}-\log\left(u\sigma^{2}\right)$ is convex and attains its minimum at $u=1/\sigma^{2}$. Evaluating Equation (A6) with these values, we see that the optimal two-moment inequality can be expressed as

$$\begin{array}{cc} \Delta_{r}\left(X\right) & =2\theta \left(\frac{r}{2(1-r)}\right)-\theta \left(\frac{r}{1-r}\right)+\frac{1}{2}\log\left(e\;r^{\frac{1}{1-r}}\right). \end{array}$$

By Equation (A4), this expression is equivalent to Equation (A1). Moreover, the fact that $\Delta_{r}\left(X\right)$ decreases to zero as *r* increases to one follows from the fact that θ(x) decreases to zero and *x* increases to infinity.

Next, we express the gap in terms of the pair (p,q). Comparing the difference between $\Delta_{r}\left(X;p,q\right)$ and $\Delta_{r}\left(X\right)$ leads to

$$\begin{array}{cc} \Delta_{r}\left(X;p,q\right) & =\Delta_{r}\left(X\right)+\frac{1}{2}\varphi \left(\frac{r\lambda (1-\lambda )}{1-r}{\left(q-p\right)}^{2}\sigma^{2}\right)+\theta \left(\frac{r\lambda }{1\;-\;r}\right)+\theta \left(\frac{r(1\;-\;\lambda )}{1-r}\right)-2\theta \left(\frac{r}{2(1\;-\;r)}\right), \end{array}$$

where φ(x)=x−log(x)−1. In particular, if p=0, then we obtain the simplified expression

$$\begin{array}{cc} \Delta_{r}\left(X;0,q\right) & =\Delta_{r}\left(X\right)+\frac{1}{2}\varphi \left(\left(q-\frac{1-r}{r}\right)\sigma^{2}\right)+\theta \left(\frac{r}{1-r}-\frac{1}{q}\right)+\theta \left(\frac{1}{q}\right)-2\theta \left(\frac{r}{2(1\;-\;r)}\right). \end{array}$$

This characterization shows that the gap of the optimal one-moment inequality ${\tilde{\Delta }}_{r}\left(X\right)$ increases to infinity in the limit as either $\sigma^{2}\to 0$ or $\sigma^{2}\to \infty$.

### Appendix B.2. Multivariate Gaussian Distribution

Let $Y∼N(0,I_{n})$ be an *n*-dimensional Gaussian vector and consider the parametrization

$$\begin{array}{cc} p & =\frac{1-r}{r}-\frac{1-\lambda }{r}\sqrt{\frac{2(1-r)\;z}{\lambda (1-\lambda )\;n}}\\ q & =\frac{1-r}{r}+\frac{\lambda }{r}\sqrt{\frac{2(1-r)\;z}{\lambda (1-\lambda )\;n}}. \end{array}$$

where λ∈(0,1) and z∈(0,∞). We can write

$$\begin{array}{cc} \log\omega (S_{Y}) & =\frac{n}{2}\log\pi -\log\left(\frac{n}{2}\right)-\log\Gamma \left(\frac{n}{2}\right)\\ \psi_{r}\left(p,q\right) & =\tilde{B}\left(\frac{r\lambda }{1-r},\frac{r(1-\lambda )}{1-r}\right)\sqrt{\frac{r\lambda (1-\lambda )}{\left(1-r\right)}}\sqrt{\frac{nr}{2z}}. \end{array}$$

Furthermore, if

(A7) $$\begin{array}{c} \left(1-\lambda \right)\sqrt{\frac{2(1-r)z}{\lambda (1-\lambda )n}}<1, \end{array}$$

then $L_{r}{(∥Y∥}^{n};p,q)$ is finite and is given by

$$\begin{array}{cc} L_{r}{(∥Y∥}^{n};p,q)\; & =Q_{r,n}\left(\lambda ,z\right)+\frac{n}{2}\log2+\frac{r}{1-r}\left[\log\Gamma \left(\frac{n}{2r}\right)-\log\Gamma \left(\frac{n}{2}\right)\right], \end{array}$$

where

(A8) $$\begin{array}{cc} Q_{r,n}\left(\lambda ,z\right)\; & =\frac{r\lambda }{1-r}\log\Gamma \left(\frac{n}{2r}-\frac{1-\lambda }{r}\sqrt{\frac{(1-r)nz}{2\lambda (1-\lambda )}}\;\right)+\frac{r(1-\lambda )}{1-r}\log\Gamma \left(\frac{n}{2r}+\frac{\lambda }{r}\sqrt{\frac{(1-r)nz}{2\lambda (1-\lambda )}}\;\right)\\ \; & \;-\frac{r}{1-r}\log\Gamma \left(\frac{n}{2r}\right). \end{array}$$

Here, we note that the scaling in Equation (21) corresponds to λ=1/2 and z=n/(n+1), and thus the condition Inequality (A7) is satisfied for all n≥1. Combining the above expressions and then using Equations (A1) and (A4) leads to

(A9) $$\begin{array}{cc} \Delta_{r}\left(Y;p,q\right) & =\theta \left(\frac{r\lambda }{1\;-\;r}\right)+\theta \left(\frac{r(1\;-\;\lambda )}{1-r}\right)-\theta \left(\frac{r}{1\;-\;r}\right)+Q_{r,n}\left(z,\lambda \right)-\frac{1}{2}\log z-\frac{1}{2}\log\left(r^{\frac{1}{r-1}}\right)\\ \; & \;+\frac{r}{1-r}\theta \left(\frac{n}{2r}\right)-\frac{1}{1-r}\theta \left(\frac{n}{2}\right). \end{array}$$

Next, we study some properties of $Q_{r,n}\left(\lambda ,z\right)$. By Equation (A1), the logarithm of the gamma function can be expressed as the sum of convex functions:

$$\begin{array}{c} \log\Gamma \left(x\right)=\varphi \left(x\right)+\frac{1}{2}\log\left(\frac{1}{x}\right)+\frac{1}{2}\log\left(2\pi \right)-1+\theta \left(x\right), \end{array}$$

where φ(x)=xlogx+1−x. Starting with the definition of Q(λ,z) and then using Jensen’s inequality yields

$$\begin{array}{cc} Q_{r,n}\left(z,\lambda \right)\; & \geq \frac{r\lambda }{1-r}\varphi \left(\frac{n}{2r}-\frac{1-\lambda }{r}\sqrt{\frac{(1-r)nz}{2\lambda (1-\lambda )}}\;\right)\\ \; & \;+\frac{r(1-\lambda )}{1-r}\varphi \left(\frac{n}{2r}+\frac{\lambda }{r}\sqrt{\frac{(1-r)nz}{2\lambda (1-\lambda )}}\;\right)-\frac{r}{1-r}\varphi \left(\frac{n}{2r}\right)\\ \; & =\frac{\lambda }{a}\varphi \left(1-\sqrt{\left(\frac{1-\lambda }{\lambda }\right)az}\right)+\frac{\left(1\;-\;\lambda \right)}{a}\varphi \left(1+\sqrt{\left(\frac{\lambda }{1-\lambda }\right)az}\right), \end{array}$$

where a=2(1−r)/n. Using the inequality $\varphi \left(x\right)\geq \left(3/2\right){\left(x-1\right)}^{2}/\left(x+2\right)$ leads to

(A10) $$\begin{array}{cc} Q_{r,n}\left(\lambda ,z\right)\; & \geq \frac{z}{2}{\left[\left(1-\sqrt{\left(\frac{1-\lambda }{\lambda }\right)bz}\right)\left(1+\sqrt{\left(\frac{\lambda }{1-\lambda }\right)bz}\right)\right]}^{-1}\\ \; & \geq \frac{z}{2}{\left(1+\sqrt{\left(\frac{\lambda }{1-\lambda }\right)b\;z}\right)}^{-1}, \end{array}$$

where b=2(1−r)/(9n).

Observe that the right-hand side of Inequality (A10) converges to z/2 as *n* increases to infinity. It turns out this limiting behavior is tight. Using Equation (A1), it is straightforward to show that $Q_{n}\left(\lambda ,z\right)$ converges pointwise to z/2 as *n* increases to infinity—that is,

(A11) $$\begin{array}{c} \lim_{n\to \infty }Q_{r,n}\left(\lambda ,z\right)=\frac{1}{2}z, \end{array}$$

for any fixed pair (λ,z)∈(0,1)×(0,∞).

### Appendix B.3. Proof of Proposition 6

Let D=(0,1)×(0,∞). For fixed r∈(0,1), we use $Q_{n}\left(\lambda ,z\right)$ to denote the function $Q_{r,n}\left(\lambda ,z\right)$ defined in Equation (A8) and we use $G_{n}\left(\lambda ,z\right)$ to denote the right-hand side of Equation (A9). These functions are defined to be equal to positive infinity for any pair (λ,z)∈D such that Inequality (A7) does not hold.

Note that the terms θ(n/(2r)) and θ(n/2) converge to zero in the limit as *n* increases to infinity. In conjunction with Equation (A11), this shows that $G_{n}\left(\lambda ,z\right)$ converges pointwise to a limit G(λ,z) given by

$$\begin{array}{cc} G(\lambda ,z) & =\theta \left(\frac{r\lambda }{1\;-\;r}\right)+\theta \left(\frac{r(1\;-\;\lambda )}{1-r}\right)-\theta \left(\frac{r}{1\;-\;r}\right)+\frac{1}{2}z-\frac{1}{2}\log\left(z\right)-\frac{1}{2}\log\left(r^{\frac{1}{r-1}}\right). \end{array}$$

At this point, the correspondence with the log-normal distribution can be seen from the fact that G(λ,z) is equal to the right-hand side of Equation (A6) evaluated with $u\sigma^{2}=z$.

To show that the gap corresponding to the log-normal distribution provides an upper bound on the limit, we use

(A12) $$\begin{array}{cc} \underset{n\to \infty }{lim sup}\Delta_{r}\left(Y\right) & =\underset{n\to \infty }{lim sup}\underset{(\lambda ,z)\in D}{\inf}G_{n}\left(\lambda ,z\right)\\ \; & \leq \underset{(\lambda ,z)\in D}{\inf}\underset{n\to \infty }{lim sup}G_{n}\left(\lambda ,z\right)\\ \; & =\underset{(\lambda ,z)\in D}{\inf}G\left(\lambda ,z\right)\\ \; & =\Delta_{r}\left(X\right). \end{array}$$

Here, the last equality follows from the analysis in Appendix B.1, which shows that the minimum of G(λ,z) is a attained at λ=1/2 and z=1.

To prove the lower bound requires a bit more work. Fix any ϵ∈(0,1) and let $D_{\epsilon }=\left(0,1-\epsilon \right]\times \left(0,\infty \right)$. Using the lower bound on $Q_{n}\left(\lambda ,z\right)$ given in Inequality (A10), it can be verified that

$$\begin{array}{c} \underset{n\to \infty }{lim inf}\underset{\left(\lambda ,z\right)\in D_{\epsilon }}{\inf}\left[Q_{r,n}\left(z,\lambda \right)-\frac{1}{2}\log z\right]\geq \frac{1}{2}. \end{array}$$

Consequently, we have

(A13) $$\begin{array}{cc} \underset{n\to \infty }{lim inf}\underset{\left(\lambda ,z\right)\in D_{\epsilon }}{\inf}G_{n}\left(\lambda ,z\right)\; & =\underset{\left(\lambda ,z\right)\in D_{\epsilon }}{\inf}G\left(\lambda ,z\right)\geq \Delta_{r}\left(X\right). \end{array}$$

To complete the proof we will show that for any sequence $\lambda_{n}$ that converges to one as *n* increases to infinity, we have

(A14) $$\begin{array}{c} \underset{n\to \infty }{lim inf}\underset{z\in (0,\infty )}{\inf}G_{n}\left(\lambda_{n},z\right)=\infty . \end{array}$$

To see why this is the case, note that by Equation (A4) and Inequality (A5),

$$\begin{array}{c} \theta \left(\frac{r\lambda }{1-r}\right)+\theta \left(\frac{r(1-\lambda )}{1-r}\right)-\theta \left(\frac{r}{1-r}\right)\geq \frac{1}{2}\log\left(\frac{1-r}{2\pi r\lambda (1\;-\;\lambda )}\right). \end{array}$$

Therefore, we can write

(A15) $$\begin{array}{c} G_{n}\left(\lambda ,z\right)\geq Q_{n}\left(\lambda ,z\right)-\frac{1}{2}\log\left(\lambda (1-\lambda )z\right)+c_{n}, \end{array}$$

where $c_{n}$ is bounded uniformly for all *n*. Making the substitution u=λ(1−λ)z, we obtain

$$\begin{array}{cc} \underset{z>0}{\inf}G_{n}\left(\lambda ,z\right) & \geq \underset{u>0}{\inf}\left[Q_{n}\left(\lambda ,\frac{u}{\lambda (1-\lambda )}\right)-\frac{1}{2}\log u\right]+c_{n}. \end{array}$$

Next, let $b_{n}=2\left(1-r\right)/\left(9n\right)$. The lower bound in Inequality (A10) leads to

(A16) $$\begin{array}{cc} \underset{u>0}{\inf}\left[Q_{n}\left(\lambda ,\frac{u}{\lambda (1-\lambda )}\right)-\frac{1}{2}\log u\right]\; & \geq \underset{u>0}{\inf}\left[\frac{u}{2\lambda }\left(\frac{1}{1-\lambda +\sqrt{b_{n}u}}\right)-\frac{1}{2}\log u\right]. \end{array}$$

The limiting behavior in Equation (A14) can now be seen as a consequence of Inequality (A15) and the fact that, for any sequence $\lambda_{n}$ converging to one, the right-hand side of Inequality (A16) increases without bound as *n* increases. Combining Inequality (A12), Inequality (A13), and Equation (A14) establishes that the large *n* limit of $\Delta_{r}\left(Y\right)$ exists and is equal to $\Delta_{r}\left(X\right)$. This concludes the proof of Proposition 6.

### Appendix B.4. Proof of Inequality (uid39)

Given any λ∈(0,1) and u∈(0,∞) let

$$\begin{array}{c} p\left(r\right)=\frac{1-r}{r}-\sqrt{\frac{1-r}{r}\left(\frac{1-\lambda }{\lambda }\right)u}\\ q\left(r\right)=\frac{1-r}{r}+\sqrt{\frac{1-r}{r}\left(\frac{\lambda }{1-\lambda }\right)u}. \end{array}$$

We need the following results, which characterize the terms in Proposition 4 in the limit as *r* increases to one.

**Lemma** **A1.**
*The function $\psi_{r}\left(p\left(r\right),q\left(r\right)\right)$ satisfies*

$$\lim_{r\to 1}\psi_{r}\left(p\left(r\right),q\left(r\right)\right)=\sqrt{\frac{2\pi }{u}}.$$

**Proof.** Starting with Equation (A4), we can write

$$\begin{array}{c} \psi_{r}\left(p,q\right)=\frac{1}{q-p}\sqrt{\frac{2\pi (1-r)}{r\lambda (1-\lambda }}\exp\left(\theta \left(\frac{r\lambda }{1\;-\;r}\right)+\theta \left(\frac{r(1\;-\;\lambda )}{1-r}\right)-\theta \left(\frac{r}{1\;-\;r}\right)\right). \end{array}$$

As *r* converges to one, the terms in the exponent converge to zero. Note that $q\left(r\right)-p\left(r\right)=\sqrt{r\lambda (1-\lambda )/(1-r)}$ completes the proof. □

**Lemma** **A2.**
*If X is a random variable such that $s\mapsto E\left[{\left|X\right|}^{s}\right]$ is finite in a neighborhood of zero, then $E\left[\log(X)\right]$ and Var(log(X)) are finite, and*

$$\lim_{r\to 1}L_{r}\left(X;p\left(r\right),q\left(r\right)\right)=E\left[\log|X|\right]+\frac{u}{2}\mathrm{Var}\left(\log|X|\right).$$

**Proof.** Let $\Lambda \left(s\right)=\log\left(E\left[{\left|X\right|}^{s}\right]\right)$. The assumption that $E\left[{\left|X\right|}^{s}\right]$ is finite in a neighborhood of zero means that $E\left[{\left(\log|X|\right)}^{m}\right]$ is finite for all positive integers *m*, and thus Λ(s) is real analytic in a neighborhood of zero. Hence, there exist constants δ>0 and C<∞, depending on the distribution of *X*, such that

$$\left|\Lambda \left(s\right)-as+bs^{2}\right|\leq C\;{\left|s\right|}^{3},\;\mathrm{for}\;\mathrm{all}\;|s|\leq \delta ,$$

where $a=E\left[\log|X|\right]$ and $b=\frac{1}{2}\mathrm{Var}\left(|X|\right)$. Consequently, for all *r* such that 1−δ<p(r)<(1−r)/r<q(r)<1+δ, it follows that

$$\begin{array}{cc} \left|L_{r}\left(X;p\left(r\right),q\left(r\right)\right)-a-\left(\frac{1-r}{r}+u\right)b\right|\; & \;\leq C\frac{r}{1-r}\left({\lambda |p\left(r\right)|}^{3}+\left(1-\lambda \right){\left|q\left(r\right)\right|}^{3}\right). \end{array}$$

Taking the limit as *r* increases to one completes the proof. □

We are now ready to prove Inequality (24). Combining Proposition 4 with Lemma A1 and Lemma A2 yields

$$\begin{array}{cc} \underset{r\to \infty }{lim sup}h_{r}\left(X\right) & \leq \frac{1}{2}\log\left(\frac{2\pi }{u}\right)+E\left[\log X\right]+\frac{u}{2}\mathrm{Var}\left(\log X\right). \end{array}$$

The stated inequality follows from evaluating the right-hand side with u=1/Var(logX), recalling that h(X) corresponds to the limit of $h_{r}\left(X\right)$ as *r* increases to one.

## Appendix C. Proof of Proposition 7

The function $\kappa :\left(0,1\right]\to R_{+}$ can be expressed as

(A17) $$\begin{array}{c} \kappa \left(t\right)=\underset{u\in (0,\infty )}{\sup}\rho_{t}\left(u\right), \end{array}$$

where $\rho_{t}\left(u\right)=\log\left(1+u\right)/u^{t}$. For t=1, the bound log(1+u)≤u implies that $\rho_{1}\left(u\right)\leq 1$. Noting that $\lim_{u\to 0}\rho_{1}\left(u\right)=1$, we conclude that κ(1)=1.

Next, we consider the case t∈(0,1). The function $\rho_{t}$ is continuously differentiable on (0,∞) with

(A18) $$\begin{array}{c} \mathrm{sgn}\left(\rho_{t}^{\prime }\left(u\right)\right)=\mathrm{sgn}\left(u-t(1+u)\log(1+u)\right). \end{array}$$

Under the assumption t∈(0,1), we see that $\rho_{t}\left(u\right)$ is increasing for all *u* sufficiently close to zero and decreasing for all *u* sufficiently large, and thus the supremum is attained at a stationary point of $\rho_{t}\left(u\right)$ on (0,∞). Making the substitution w=log(1+u)−1/t leads to

$$\begin{array}{c} \rho_{t}^{\prime }\left(u\right)=0\;\Leftrightarrow \;we^{w}=-\frac{1}{t}e^{-\frac{1}{t}}. \end{array}$$

For t∈(0,1), it follows that $-\frac{1}{t}e^{-\frac{1}{t}}\in \left(-e^{-1},0\right)$, and thus $\rho_{t}^{\prime }\left(u\right)$ has a unique root that can be expressed as

$$u_{t}^{*}=\exp\left(W\left(-\frac{1}{t}\exp\left(-\frac{1}{t}\right)\right)+\frac{1}{t}\right)-1,$$

where Lambert’s function W(z) is the solution to the equation $z=we^{w}$ on the interval on [−1,∞).

**Lemma** **A3.**
*The function g(t)=tκ(t) is strictly increasing on (0,1] with $\lim_{t\to 0}g\left(t\right)=1/e$ and g(1)=1.*

**Proof.** The fact that g(1)=1 follows from κ(1)=1. By the envelope theorem [37], the derivative of g(t) can be expressed as

$$g^{\prime }\left(t\right)=\frac{d}{dt}\;t\rho_{t}\left(u\right){|}_{u=u_{t}^{*}}=\frac{\log(1+u_{t}^{*})}{{\left(u_{t}^{*}\right)}^{t}}-t\log\left(u_{t}^{*}\right)\frac{\log(1+u_{t}^{*})}{{\left(u_{t}^{*}\right)}^{t}}$$

In view of Equation (A18), it follows that $\rho_{t}^{\prime }\left(u_{t}^{*}\right)=0$ can be expressed equivalently as

(A19) $$\frac{u_{t}^{*}}{\left(1+u_{t}^{*}\right)\log\left(1+u_{t}^{*}\right)}=t,$$

and thus

(A20) $$\begin{array}{c} \mathrm{sgn}\left(g^{\prime }\left(t\right)\right)=\mathrm{sgn}\left(1-\frac{u_{t}^{*}\log u_{t}^{*}}{\left(1+u_{t}^{*}\right)\log\left(1+u_{t}^{*}\right)}\right). \end{array}$$

Noting that ulogu<(1+u)log(1+u) for all u∈(0,∞), it follows that $g^{\prime }\left(t\right)>0$ is strictly positive, and thus g(t) is strictly increasing. To prove the small *t* limit, we use Equation (A19) to write

(A21) $$\begin{array}{c} \log\left(g\left(t\right)\right)=\log\left(\frac{u_{t}^{*}}{1+u_{t}^{*}}\right)-\frac{u_{t}^{*}\log u_{t}^{*}}{\left(1+u_{t}^{*}\right)\log\left(1+u_{t}^{*}\right)}. \end{array}$$

Now, as *t* decreases to zero, Equation (A19) shows that $u_{t}^{*}$ increases to infinity. By Equation (A21), it then follows that log(g(t)) converges to negative one, which proves the desired limit. □
